# Efficacy of Erymicin 200 Injections for Reducing *Renibacterium salmoninarum* and Controlling Vertical Transmission in an Inland Rainbow Trout Brood Stock

**DOI:** 10.3390/pathogens9070547

**Published:** 2020-07-07

**Authors:** Eric R. Fetherman, Brad Neuschwanger, Tracy Davis, Colby L. Wells, April Kraft

**Affiliations:** 1 Colorado Parks and Wildlife, Aquatic Wildlife Research Section, 317 West Prospect Road, Fort Collins, CO 80526, USA; 2 Colorado Parks and Wildlife, Bellvue Fish Research Hatchery, 5500 West County Road 50E, Bellvue, CO 80512, USA; Brad.Neuschwanger@state.co.us (B.N.); Tracy.Davis@state.co.us (T.D.); 3 Colorado Parks and Wildlife, Aquatic Animal Health Laboratory, 122 East Edison Street, Brush, CO 80723, USA; Colby.Wells@state.co.us (C.L.W.); April.Kraft@state.co.us (A.K.)

**Keywords:** rainbow trout, *Oncorhynchus mykiss*, brood stock, *Renibacterium salmoninarum*, bacterial kidney disease, antibiotics, Erymicin 200

## Abstract

Bacterial Kidney Disease, caused by *Renibacterium salmoninarum* (*Rs*), is widespread and can cause significant mortality at most life stages in infected salmonids. *Rs* is commonly found in inland trout, which can be carriers of the bacterium. Lethal spawns can be used to control vertical transmission to progeny through the culling of eggs from infected parents, but can be costly, time-consuming, and can negatively impact important and rare brood stocks. Erymicin 200 is an Investigational New Animal Drug (INAD) intended to reduce *Rs* levels in hatchery brood stocks and control vertical transmission to progeny. We tested the efficacy of Erymicin 200 injections in a positive, hatchery-resident rainbow trout (*Oncorhynchus mykiss*) brood stock in Colorado, USA. Brood fish age two and three were injected with 25 mg per kg of body weight Erymicin 200 three times prior to spawning. Erymicin 200 was effective in reducing *Rs* to below detectable levels in treated fish. However, both negative treated and control brood fish produced positive progeny, suggesting that Erymicin 200 did not prevent the vertical transmission of *Rs.*

## 1. Introduction

Bacterial Kidney Disease (BKD), caused by *Renibacterium salmoninarum* (*Rs*), is widespread worldwide where wild or cultured salmonids are present, and can cause significant mortality at most life stages [1]. *Rs* is an intracellular, gram-positive diplobacillus that can be transmitted both horizontally [2,3,4] and vertically [5,6]. The bacterium is known to attach to the flagellum of spermatozoa which is lost at egg fertilization [5,6,7,8], and eggs fertilized with milt-containing *Rs* have subsequently tested negative for *Rs* [6]. As such, vertical transmission is suspected to be primarily maternal, originating and developing through ovarian tissues [5,9]. Iodophor disinfection does not eliminate intraovum infections [5,6], making traditional egg disinfection techniques ineffective for preventing vertical transmission during hatchery spawning. Additionally, prophylactic antibiotic-medicated feed treatments are not entirely protective [1,10,11,12], and reduced antibiotic susceptibility exists in *Rs* [13,14]. *Rs* is commonly found in wild and hatchery-reared inland trout, which can be carriers of the bacterium. Inland trout species are more resistant to BKD than anadromous salmonids [15], and resistance to BKD varies among species, with brook trout (*Salvelinus fontinalis*) showing more susceptibility to *Rs* infection than brown trout (*Salmo trutta*) or rainbow trout (*Oncorhynchus mykiss*) [16].

*Rs* outbreaks were a major problem in Colorado’s hatchery system in the 1950s and 1960s, often related to water quality, and high-density and high-stress rearing conditions. Although outbreaks were reduced by changing rearing standards, *Rs* was detected 16 times at state and federal fish hatcheries between 1970 and 1996, from which millions of fish were stocked into all major river drainages in Colorado [17]. *Rs* was not detected in Colorado hatcheries and brood stocks during routine health inspections for 18 years. However, one federal and five state hatcheries have tested positive for *Rs* in Colorado since 2015. 

*Rs* can inadvertently be introduced to the hatchery rearing environment during wild spawning operations via vertical transmission routes [5,6], the suspected introduction route to the Colorado Parks and Wildlife (CPW) Glenwood Springs Hatchery (GSH; Glenwood Springs, CO, USA) in 2015. Cutthroat trout (*Oncorhynchus clarkii*) originating from wild spawn operations, were incorporated into the upper-most raceway of GSH after testing negative for *Rs* following appropriate rearing time in an isolation facility, and subsequently tested positive for *Rs* during routine annual health inspections. Given the potential for horizontal transmission to other fish on the unit and state regulations restricting movement and stocking of *Rs*-infected fish, GSH was depopulated to eliminate the bacteria. Depopulation costs exceeded 2.1 M USD, and resulted in the loss of thousands of pounds of sport and brood fish.

In 2015 and 2016, two other CPW hatcheries housing important and irreplaceable brood stocks tested positive for *Rs*, and therefore depopulation was not an option. Cull spawning and screening of fish to prevent *Rs-*infected eggs from becoming part of production have successfully reduced *Rs* in hatchery brood stocks [8,18]. As such, CPW implemented a lethal spawning procedure at both units during which all spawned adults were culled and tested for *Rs*. Eggs originating from parents that tested positive for *Rs* were also culled. Although culling operations can continue in perpetuity to maintain low to non-existent levels of infection in offspring [8], they can be costly, time-consuming, difficult to maintain, and result in the loss of fish after a single spawn.

As an alternative, erythromycin injections have been shown to minimize vertical transmission of *Rs* through incorporation of the antibiotic in the eggs [19,20,21,22,23,24] and progeny [22,23] originating from injected brood fish. A macrolide antibiotic isolated from *Streptomyces arythraus* [25], the bacteriostatic [25,26] and bactericidal [25] properties of erythromycin are enacted by targeting the protein synthesis of the 50 S subunit of the ribosome of gram-positive bacteria [26]. The side effects of erythromycin injections include ascites [27,28,29] as well as drug-induced hemolytic anemia/hyperbilirubinemia [28], and mortality has been associated with toxicity of the drug carrier [27,29]. However, Moffit and Kiryu [30] demonstrated that dosages of up to 40 mg erythromycin per kg can be repeatedly injected interperitoneally with few drug-related negative effects.

A relatively low-cost, real-world experiment, designed such that it could be executed using normal hatchery biosecurity and daily feeding, spawning, and cleaning procedures was therefore conducted at the CPW Bellvue Fish Research Hatchery (BFRH; Bellvue, CO, USA) to examine the use of erythromycin (Erymicin 200) injections to control *Rs* in a rainbow trout brood stock. The objective was to determine the efficacy of Erymicin 200 injections to reduce or minimize *Rs* levels in positive brood fish to control and/or prevent the vertical transmission of *Rs* to progeny using the maximum dosage allowed under the Aquatic Animal Drug Approval Partnership Program (AADAP) Investigational New Animal Drug (INAD) study #12–781. We hypothesized that fewer brood fish treated with Erymicin 200 would test positive for *Rs* compared to untreated control fish, thereby reducing vertical transmission. As a result, we expected fewer progeny originating from treated adults to test positive for *Rs* relative to those originating from control adults. Hypotheses were evaluated using single-round polymerase chain reaction (PCR) to enumerate adult and progeny fish testing positive or negative for *Rs*.

## 2. Results

**Figure 1:**
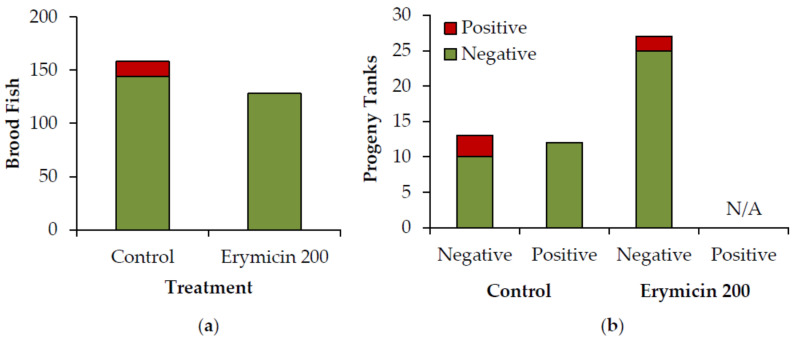
(**a**) Number of brood fish that tested positive for *Rs* by treatment; (**b**) Number of progeny tanks that tested positive for *Rs* by treatment and status (positive or negative) of the parental brood fish. The presence of *Rs* was assessed using homogenized kidney tissue (brood fish) or five-fish pooled, homogenized whole fish tissue (progeny) and single-round PCR.

The presence of *Rs* was evaluated using homogenized kidney tissue samples and single-round PCR in both rainbow trout brood fish injected with a dose of 25 mg Erymicin 200 per kg body weight three times prior to spawning (treated) and untreated control fish. Erymicin 200 injections resulted in significantly fewer treated fish testing positive for *Rs* compared to controls (Fisher’s exact test, *p* < 0.001). No brood fish tested positive for *Rs* after being treated with Erymicin 200. In comparison, 91% of control fish tested negative, whereas 9%, comprised of male and female fish of two age classes, tested positive for *Rs* (Figure 1).

Upon swim-up of the progeny, 12 five-fish pooled, whole fish tissue samples were collected from each tank, homogenized, and tested for *Rs* using single-round PCR. Injections of Erymicin 200 did not prevent vertical transmission from brood fish to progeny, with similar numbers of progeny tanks testing positive for *Rs* originating from treated and control brood fish (Fisher’s exact test, *p* = 0.66; Figure 1). The prevalence of *Rs* in progeny tanks originating from *Rs*-negative treated adults was 8.3 ± 0.0% (mean ± SE), compared to 16.6 ± 8.3% in progeny tanks originating from *Rs*-negative control adults. None of the 12 progeny tanks originating from *Rs*-positive control adults tested positive for *Rs* (Figure 1).

## 3. Discussion

Other studies have demonstrated the effectiveness of erythromycin injections, albeit with different formulations, in reducing *Rs* vertical transmission rates in migratory salmonid species with high infection prevalence in the Pacific Northwest, USA [19,20,21,22]. This is one of the first studies to evaluate the efficacy of erythromycin in reducing *Rs* levels and thereby vertical transmission in a hatchery-resident population of inland salmonids with low infection prevalence. As we hypothesized, Erymicin 200 effectively reduced *Rs* to below detectable levels in rainbow trout brood fish. However, similar to previous research [22], the injections did not eliminate *Rs* in the brood fish, and vertical transmission of *Rs* still occurred.

A number of factors can affect erythromycin concentration in brood fish, eggs, and progeny, including concentration of erythromycin used [24], timing of injections in relation to spawn timing [19,20,22], and method of delivery [22]. We used the highest Erymicin 200 concentration and maximum number of treatments allowed under the INAD study protocol. Although concentrations as low as 10 to 20 mg/kg [19,20,21,22] have been effective for achieving the in vitro minimum inhibitory concentration of 0.3 μg/mL in eggs [20], Haukenes and Moffitt [24] found that 40 mg/kg resulted in higher concentrations of erythromycin in the kidneys of female brood fish and vitellin of the eggs 14–16 days after injections occurred. Erythromycin concentrations were not measured in this study. However, concentrations of up to 0.6 ppm have been shown to persist in the eggs 30 to 60 days post-injection [19], after which time the minimum inhibitory concentration may still be present 68–75 days post-injection [21], but could be reduced and not detectable by 70 days post-injection [20]. Although the fish in our study were first spawned 35 days post-injection, the final group was spawned 63 days post-injection, outside the effective range for erythromycin retention [19,20] and at a time in the spawning period when *Rs* infection prevalence was likely higher [31]. Indeed, three positive progeny groups were spawned the last week of the study. Additionally, two of the positive progeny groups originating from treated adults were spawned using three-year-old females. Upon inspection of a mortality that occurred during the third injection period, it appeared that the 6 mm needle was just long enough to enter the intraperitoneal cavity of the three-year-old fish. This may have resulted in intramuscular injections in some fish, which are usually administered in the dorsal sinus [22] rather than the abdominal wall. These factors may have affected the efficacy of Erymicin 200 injections in our study.

A number of detection methods can be used for *Rs*, but to date, none have demonstrated high analytical and diagnostic performance characteristics over the others [32]. Detection methods can also vary based on the progression of infection within the fish [33]. Single-round PCR was the only diagnostic tool used to determine the presence of *Rs* DNA in brood fish and progeny at BFRH in this study, which may not have been sensitive enough to detect trace levels of infection. Quantitative PCR [34] and nested PCR [35] techniques can be more sensitive for *Rs* detection, resulting in fewer false negatives than conventional serological tests, and nested PCR is recommended for *Rs* confirmation by the American Fisheries Society Fish Health Section (AFS-FHS) [36]. However, nested PCR is more laborious and prone to potential laboratory contamination than conventional PCR [37]. The single-round PCR technique used by CPW was developed using the AFS-FHS nested PCR protocol [36] as a platform, and in-house validation efforts suggest that qPCR and conventional PCR techniques show similar sensitivity to *Rs* detection. As recommended by the AFS-FHS [36], kidney tissue was the primary tissue used for the detection of *Rs* in the brood fish. Because the bacterium may not be evenly distributed in the kidney tissue in the early stages of infection [38], and the eggs can test positive for *Rs* when the kidney tissue is negative [39,40], other tissues such as the liver [41] or spleen [41,42] may be useful for determining *Rs* presence in hatchery-reared fish. Additionally, ovarian fluid has a high *Rs* detection rate in Chinook salmon [43], detecting as few as 28 bacterial cells/ml [44], however, it is inadequate for *Rs* detection in Atlantic salmon [45], suggesting that efficacy may be species-specific. Future studies would benefit from using a number of diagnostic methods [46], complimentary techniques such as enzyme-linked immunosorbent assay (ELISA), culture and other well-validated methods [37], and multiple tissue samples to quantify prevalence and ensure negative status of brood fish and/or progeny.

Our study shows that Erymicin 200 injections, though not completely effective for elimination of *Rs*, are a useful management tool for reducing *Rs* in hatchery brood fish and vertical transmission to progeny. However, although not observed in all situations [47], widespread antibiotic use in aquaculture can lead to the development of antibiotic resistance. Resistance to erythromycin has been demonstrated in *Aeromonas hydrophila* [48], *Flavobacterium columnare* [49], *Vibrio* spp. [50], and *Rs* [13,14]. Similar to terrestrial agriculture, the development of antibiotic resistance in aquaculture pathogens has potential for human health consequences [51] and limiting antibiotic use may slow the development of such resistance. Therefore, programs seeking to eliminate *Rs* without depopulating the hatchery unit could benefit from a combination of management strategies, including erythromycin injections, cull spawning, enhancement of hatchery biosecurity measures [18], and using multiple testing strategies or testing a variety of tissues to achieve that goal.

## 4. Materials and Methods 

The BFRH consists of a hatchery building, two isolation buildings, and covered outdoor raceways. The unit is supplied with water from an on-site well that maintains the following water quality parameters year round: (a) temperature 12 °C; (b) dissolved oxygen 9 mg/L; (c) pH 7.0; and, (d) water hardness (CaCO_3_) 120 mg/L. The well has no surface water connection or known biota, such as a population of fish, that could be a source of *Rs*. Although the water source has never been tested specifically for *Rs*, fish within the hatchery and isolation buildings have previously tested negative, providing confidence that the water was not a source of *Rs* during this experiment. Water in the hatchery and isolation buildings, where eggs and small fish are reared, is first-use. In the isolation buildings, water is run to a septic drain after use. Water from the hatchery building is secondarily used in outdoor raceways; however, during cleaning, tank water is run to a septic drain rather than being retained for use on the unit. Therefore, only clean, second-use water is supplied to the outdoor raceways where the brood stocks are maintained.

The German Rainbow (GR) brood stock at BFRH is used to produce *M. cerebralis*-resistant GR and GR-cross fish for stocking and research purposes. The GR spawn, following a bell curve distribution between mid-November and mid-December, and weekly spawns within this period, are used to collect gametes from newly ripened fish. Cull spawning was implemented following the detection of *Rs* in 2016 to reduce vertical transmission and prevalence of *Rs* on the unit. Two- and three-year-old GR brood fish of both sexes have since tested positive for Rs using direct fluorescent antibody test (DFAT) and single-round PCR, with prevalence ranging from 0% to 10.2% annually, although clinical signs of disease have been rare. 

**Figure 2:**
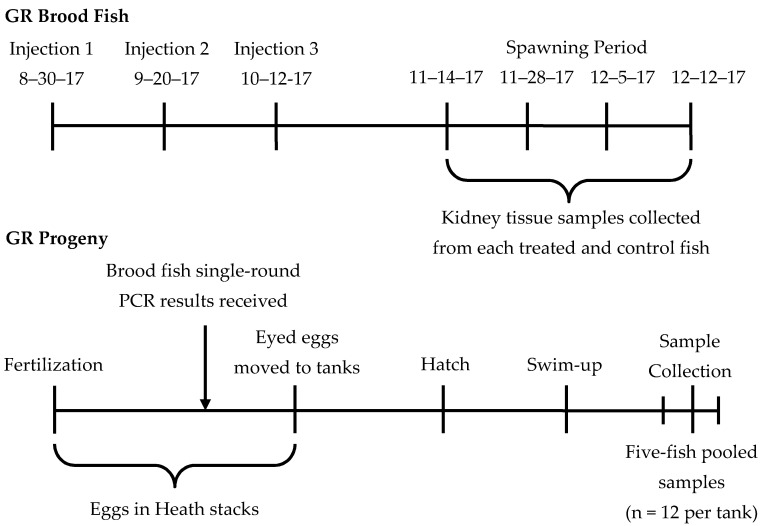
German Rainbow (GR) brood fish injection, spawning, and kidney tissue sample collection timeline and GR progeny timeline from fertilization on a given spawn date to collection of five-fish pooled samples 49 ± 4 days post-fertilization. The presence of *Rs* was assessed in the GR brood fish and progeny using single-round PCR.

The experimental design and injection procedures were approved by the AADAP program prior to initiation (study approval #12-781-17-016), and all injection, spawning, and tissue collection procedures were either conducted by CPW aquatic veterinarians or with their oversight. Two- and three-year-old GR brood fish (n = 200) were treated with three injections of Erymicin 200 Injection (Syndel; Ferndale, Washington; Lot #25865) prior to being spawned. Prior to injection, fish were anesthetized with tricane methanesulfonate (MS-222; Syndel; Ferndale, Washington) and sex was determined, if possible. Despite the lack of evidence that males contribute to the vertical transmission of *Rs* [5,6,7], both male and female brood fish were treated with Erymicin 200, in part because sex was difficult to ascertain, but also because the male contribution to vertical transmission in inland trout is unknown. All fish were weighed (average 2.0 kg) to allow administration of a standardized dose of 25 mg Erymicin 200 per kg of body weight, injected into the IP cavity through the midventral abdominal wall using a Socorex 5 cc self-refilling syringe (Socorex Isba SA; Ecublens) and 0.7 × 6 mm needle (Unimed; Lausanne). Following injection, fish were returned to the raceway to recover from anesthetization and handling. Twenty-one days passed between the first and second injections, and 22 days between the second and third injections (Figure 2). Untreated control fish (n = 334) were not handled during the injection period, but were maintained under the same conditions as the treated fish. Between the first injection and final spawn, treated and control fish were cared for following standard hatchery procedures [52], cleaned regularly using disinfected equipment between raceways, and fed roughly 0.015 kg feed (Rangen; Buhl, Idaho) per kg of fish daily.

Spawning and egg water hardening procedures occurred alongside the outdoor raceways in which the brood fish were held four times between mid-November and mid-December 2017 (Figure 2). During weekly spawning events, treated and control fish were sorted by ripeness, and groups of four to five fish were anesthetized using MS-222 to allow for handling. Single male–female egg groups were created by spawning a three-year-old female with a two-year-old male, or vice versa, and maintained separately until brood fish testing results were obtained (Figure 2). Brood fish were euthanized after being spawned, and anterior, mid-, and posterior kidney tissue samples were collected from each and stored in 70% ETOH until DNA extraction could occur, 24–48 h after collection. After fertilization, egg groups were kept in individual, labeled egg cups and water hardened in a 50 ppm iodine solution for one hour.

Before entering the hatchery, egg cups were submerged in a 100 ppm iodine bath for an additional ten minutes to prevent transporting bacteria or other pathogens into the hatchery building. Egg cups were then placed in Heath stacks and treated every two to three days until eyed with a solution of 1667 ppm formalin for 15 min at a flow of five gallons per minute to prevent fungus from killing the eggs. Upon eyeing, 19 (±2) days post-fertilization, eggs were moved to 9.5 L tanks to hatch (Figure 2). Due to space requirements, eggs originating from *Rs*-negative adults were pooled in tanks by treatment within the hatchery building, with two to three single male-female families per tank. As such, 13 control pooled-family tanks and 27 treated pooled-family tanks were maintained in the hatchery building for *Rs* progeny testing. Eggs originating from *Rs*-positive adults were moved into 9.5 L tanks located in one of the isolation buildings to prevent possible horizontal transmission of *Rs* in second-use water. Space in the isolation building was not limited, and as such, families were maintained separately by tank, resulting in 12 tanks maintained for *Rs* progeny testing. Standard hatchery biosecurity protocols were followed in both locations, namely maintaining distinct equipment for use during the study, not transferring equipment between buildings, disinfection of equipment after use in a tank, and disinfection of hands and footwear while entering and exiting the buildings, to prevent horizontal transmission among the tanks. Eggs hatched approximately 24 days post-fertilization, and swim-up occurred two weeks later, approximately 38 days post-fertilization. Eleven (±4) days after swim-up, 60 fish from each tank were collected for *Rs* testing (Figure 2). The egg sacs, heads, and tails were removed from each individual, and five whole-fish samples were pooled (n = 12 pooled samples per tank) to provide enough tissue for DNA extraction.

DNA was extracted from homogenized kidney tissue (adult) or pooled, homogenized whole-fish tissue (progeny) by the CPW Aquatic Animal Health Lab (Brush, Colorado, CO, USA) using the Qiagen DNeasy Blood and Tissue Kit (Hilden, Germany), according to kit protocol. A 25 mg tissue sample was used for extractions. DNA was eluted in 200 μL Buffer AE and stored at 2–8 °C. A positive extraction control (PEC) and a negative extraction control (NEC) were processed at the same time as the samples. The PEC consisted of *Rs*-infected kidney tissue samples originating from *Rs*-positive fish in Colorado hatcheries and confirmed via sanger sequencing. The NEC substituted molecular grade water.

Single-round PCR was used to amplify *Rs* DNA, if present. The following PCR protocol was developed, optimized, and validated by Pisces Molecular (Boulder, CO, USA), adopted by the AAHL in 1995, and has since been used regularly as a confirmatory assay. A total of 5 μL of extracted DNA was added to 45 μL of reaction mixture containing 5× Green GoTaq^®^ Flexi Buffer without Mg^2+^, 2.5 mM MgCl_2_, 0.4 mM dNTP Mix, 0.8 μM forward and reverse primer, 0.04 U/μL GoTaq^®^ Flex DNA Polymerase, and molecular grade water. All reagents for the master mix were supplied by Promega Corporation (Madison, WI, USA). The primers used for the PCR amplification reaction target the major antigen solubility gene and were Forward 5’-TTTGGGGTGGCTCCTCTTGCG-3’, PM14, and Reverse 5’-ATTGGGGATGGCGCATTATCG-3’, PM15 (Sigma-Aldrich; St. Louis, MO, USA). In addition to the PEC and NEC, a positive template control (PTC), derived from previously extracted *Rs*-infected kidney tissue samples and confirmed via sanger sequencing, and a no template control (NTC), consisting of 45 and 5 μL DNA-free water, were run. The PTC provided consistency amongst sample types and mitigated quality control concerns as DNA concentrations from unknown samples were not quantified as part of this study. Amplification was performed on an MJ Research PTC-200 Peltier Thermal Cycler (MJ Research, Inc., Waltham, MA, USA). The thermal profile for the amplification process included one cycle at 94 °C for two minutes followed by 35 cycles with the following steps: (1) 94 °C for thirty seconds, (2) 67 °C for one minute, and, (3) 72 °C for one minute and thirty seconds. The reaction was held at 4 °C until gel electrophoresis was performed. 

Amplified DNA was visualized by gel electrophoresis on an Invitrogen and 2% agarose gel using SYBR^®^ Safe DNA gel stain (Invitrogen; Carlsbad, CA, USA), viewed on a transilluminator, and the size determined via an E-Gel 1 kb Plus DNA Ladder (Invitrogen; Carlsbad, CA, USA) and compared to the positive controls. The assay was considered valid if all controls worked as expected. A fish was considered positive if the anticipated 377 base–pair product was observed, corresponding to the bands produced by the positive controls. A Fisher’s exact test was used to determine whether treatment resulted in a reduction in the number of brood fish testing positive for *Rs* at the time of spawning, relative to the control fish, and if fewer progeny originating from treated adults tested positive for *Rs*, relative to those originating from control adults.
